# Abortive Treatment of Erysipelas of the Face

**Published:** 1893-08

**Authors:** 


ABORTIVE TREATMENT OF ERYSIPELAS OF THE FACE.—A one per cent. etherial solution of sublimate should be used, applied with a small hand atomizer, throwing a forcible spray.

The more forcibly the spray is applied so much quicker will be the recovery, depending of course on the thickness of the skin and the severity of the case.

The small blisters or vesications which the sublimate may cause should not be the cause of its withdrawal, for in the smaller erysipelatous eruptions they should be encouraged rather than otherwise, on account of their beneficial effect.

In applying the spray, the central parts of the inflammatory areas should be only lightly sprayed, but much more thoroughly along the line of demarkation, as well as one to two cm. into the healthy skin. The eyelids should be only slightly moistened. Then apply compresses. One or two such applications of the sublimate should be sufficient. Those towards the last must be shorter, and parts which have been gone over once should be only lightly touched a second time. Only the boundaries and suspected places on the skin require the more energetic spraying.

Before commencing the treatment the patient should be informed that after every application of the spray he will feel a rather sharp burning pain, but which will not be any more severe than the discomfort caused by the tension of the skin from the erysipelas; also that his face will swell and small blisters or vesicles will form, which might likewise be caused by an erysipelatous inflammation. The crusts should not be removed, but allowed to drop off spontaneously.

